# Sense of ownership is linked to the speed of visuomotor adaptation in virtual reality but not to generalization, intermanual transfer, or aftereffects

**DOI:** 10.1038/s41598-025-03697-y

**Published:** 2025-05-28

**Authors:** Johanna Gerken, Opher Donchin, Thomas Abel, Susen Werner

**Affiliations:** 1 Institute of Movement and Neurosciences, German Sport University, Cologne, Germany; 2 Department of Biomedical Engineering, Ben-Gurion University of the Negev, Be’er Sheva, Israel; 3 Institute of Professional Sport Education and Sport Qualifications, German Sport University, Cologne, Germany; 4 Institute of Movement and Neurosciences, German Sport University, Am Sportpark Müngersdorf 6, 50933 Cologne, Germany

**Keywords:** Sensorimotor adaptation, Motor learning, Generalization, Intermanual transfer, Virtual reality, Explicit process, Neurophysiology, Spatial memory

## Abstract

Visuomotor adaptation, a form of motor learning in which familiar movements are adjusted to changes in visual feedback, has been widely studied in 2D settings. Testing this in virtual reality (VR) enables the exploration of 3D movements, offering a more realistic representation of motor behavior in daily tasks. While some studies have compared adaptation in 2D and 3D, none have examined the effect of visual feedback type. We compared adaptation via either a virtual hand or a cursor as feedback. The participants adapted to a visuomotor rotation while reaching in VR, and we assessed adaptation performance, spatial generalization, intermanual transfer, aftereffects, and sense of agency. Hand-based feedback facilitated faster adaptation, likely due to a stronger sense of agency, as indicated by a positive correlation between agency ratings and the adaptation rate. However, spatial generalization, intermanual transfer, and aftereffects did not differ between feedback types, suggesting that adaptation remained context-dependent and that the feedback modality did not influence the balance between explicit and implicit learning. We observed high intermanual transfer (~ 80%), driven primarily by explicit adaptation, which is consistent with previous 2D studies. These findings provide insights into visual feedback’s role in sensorimotor learning and have implications for rehabilitation and skill training in immersive environments.

**Supplementary Information:**

The online version contains supplementary material available at 10.1038/s41598-025-03697-y.

## Introduction

Sensorimotor adaptation has long been a fundamental topic in motor learning research, with significant breakthroughs emerging from studies on prism adaptation. One of the pioneering investigations in this field was the “Innsbruck Goggle Experiments,” where Theodor Erismann explored the effects of long-term prism adaptation on perception and motor execution^1^. These early experiments demonstrated that when individuals adapted to prism goggles, their motor responses adjusted accordingly, and the aftereffects of adaptation were notable. With advances in technology, these studies evolved, and researchers began employing computer-generated perturbations to investigate adaptation. These experiments introduced perturbations such as rotational perturbations of visual feedback^2–4^, commonly referred to as “visuomotor adaptation.” Additionally, force-field adaptations have been used to study motor learning under dynamic perturbations^5–7^.

While visuomotor adaptation has been extensively studied in two-dimensional (2D) settings, recent advancements in virtual reality (VR) technology have enabled more immersive, three-dimensional (3D) adaptation experiments. Head-mounted VR devices offer a strong sense of embodiment, allowing participants to perceive themselves as being physically present in a virtual environment^8–11^. Importantly, VR allows for the investigation of full 3D movements, which more closely resemble natural motor behavior. This enhances the ecological validity of the findings and improves their transferability to real-world applications, such as rehabilitation and sports science^12,13^. Studies by Ferrea et al.^14^ and Lefrançois & Messier^15^ have examined adaptation to perturbations in three planes, demonstrating that motor adaptation is feasible in 3D spaces and may depend on the axis of perturbation. Additionally, Anglin et al.^16^ reported that visuomotor adaptation in VR environments produced similar adaptation effects to those of conventional 2D settings but with a more explicit strategy. Ramos et al.^17^ reported greater aftereffects in VR than in physical prism adaptation, highlighting the potential of VR as a tool for studying adaptation. However, none of these studies systematically compared different types of visual feedback within the same virtual environment.

First, the nature of visual feedback could influence the sense of agency – the perception that one is in control of one’s actions. The sense of agency has been linked to individual differences in learnability during sensorimotor adaptation^18^. Wen et al.^18^ assessed sense of agency via a perceptual judgment task in which participants rated their control over movements, while adaptation was measured through kinematic changes in response to visuomotor perturbations. Their findings indicated that individuals with a stronger sense of agency exhibited greater adaptability. If participants experience a stronger sense of agency when seeing their own (virtual) hand compared to a cursor, this might affect their ability to adapt in our study.

Second, the type of visual feedback provided during adaptation is a critical factor influencing the underlying learning mechanisms. Different types of feedback may trigger distinct forms of learning, such as context-dependent versus context-independent learning. In prism adaptation, where individuals see their own hand, adaptation effects tend to generalize more broadly across different spatial contexts^19–21^. In this context, spatial generalization is defined as the transfer of adaptation to untrained spatial locations^15,22^. This suggests that prism adaptation may facilitate context-independent learning, where the adapted motor response is applied across multiple situations. In contrast, visuomotor adaptation, which often employs a cursor as a proxy for hand position, tends to be highly context-dependent, with learning effects being more specific to the trained condition^22,23^. Given this, the presence of hand feedback in our study might facilitate broader generalization effects.

Third, the type of visual feedback may influence whether sensory prediction errors are attributed to internal or external causes, which in turn affects the magnitude of recalibration or aftereffects. Wilke et al.^24^ demonstrated that larger aftereffects occur when individuals perceive prediction errors as self-generated rather than caused by external disturbances. Additionally, gradual perturbations tend to result in larger aftereffects than sudden perturbations^25^, and the magnitude of aftereffects is negatively correlated with an individual’s awareness of the perturbation^26,27^. This suggests that implicit adaptation processes are more dominant when aftereffects are pronounced^28–32^. However, the distinction between explicit and implicit components remains terminologically and conceptually ambiguous in the literature, particularly with regard to the role of consciousness^33^. In this study, we define explicit learning as conscious, strategic adjustments that participants can verbalize or intentionally implement. In contrast, implicit learning refers to automatic, unconscious recalibrations of motor output in response to sensory prediction errors—adjustments that occur without deliberate effort or awareness^34–36^. We adopt a behavioral operationalization: explicit adaptation is assessed by instructing participants to consciously apply learned strategies, whereas implicit adaptation is inferred from residual performance changes when participants are instructed not to compensate. Since prism adaptation is closely linked to implicit learning, the presence of visual feedback of one’s own (virtual) hand might reinforce an internal attribution of errors and enhance recalibration. However, intermanual transfer in visuomotor adaptation is often consciously mediated and associated with explicit learning mechanisms^27,37,38^. Therefore, if implicit learning dominates in conditions with hand feedback, one might predict that stronger aftereffects will be observed in these conditions, but intermanual transfer may be reduced compared with cursor-based feedback. This relationship between visual feedback type, error attribution, and learning mechanisms will be tested in our experiment.

In this study, we investigate whether visual feedback influences the adaptation process in VR by comparing adaptation effects when participants see their own (virtual) hand versus a representative cursor. We aim to address three key questions: (1) Does hand-based visual feedback enhance the sense of agency and thereby improve adaptation performance? (2) Does adaptation with hand-based visual feedback result in greater spatial generalization, thereby supporting the idea of context-independent learning? (3) Does hand-based visual feedback strengthen implicit learning mechanisms, leading to larger aftereffects but reduced intermanual transfer?

By addressing these questions, we aim to contribute to the understanding of how visual feedback modulates adaptation in virtual environments and how these insights can inform broader theories of sensorimotor learning.

## Results

This study investigated how different forms of visual feedback—either a virtual hand or a cursor—affect visuomotor adaptation in a virtual reality (VR) environment. Thirty right-handed adults (aged 18–30 years) participated and were randomly assigned to either the hand (H) or cursor (C) feedback group. Using a VR setup with wireless data gloves, participants performed center-out reaching movements. During the adaptation phase, visual feedback was rotated by 40°. To assess spatial generalization, performance was tested on untrained target directions. Intermanual transfer was evaluated using the non-dominant (left) hand. Additionally, explicit and implicit components of learning were assessed in both conditions through specific task instructions.

**Fig. 1:**
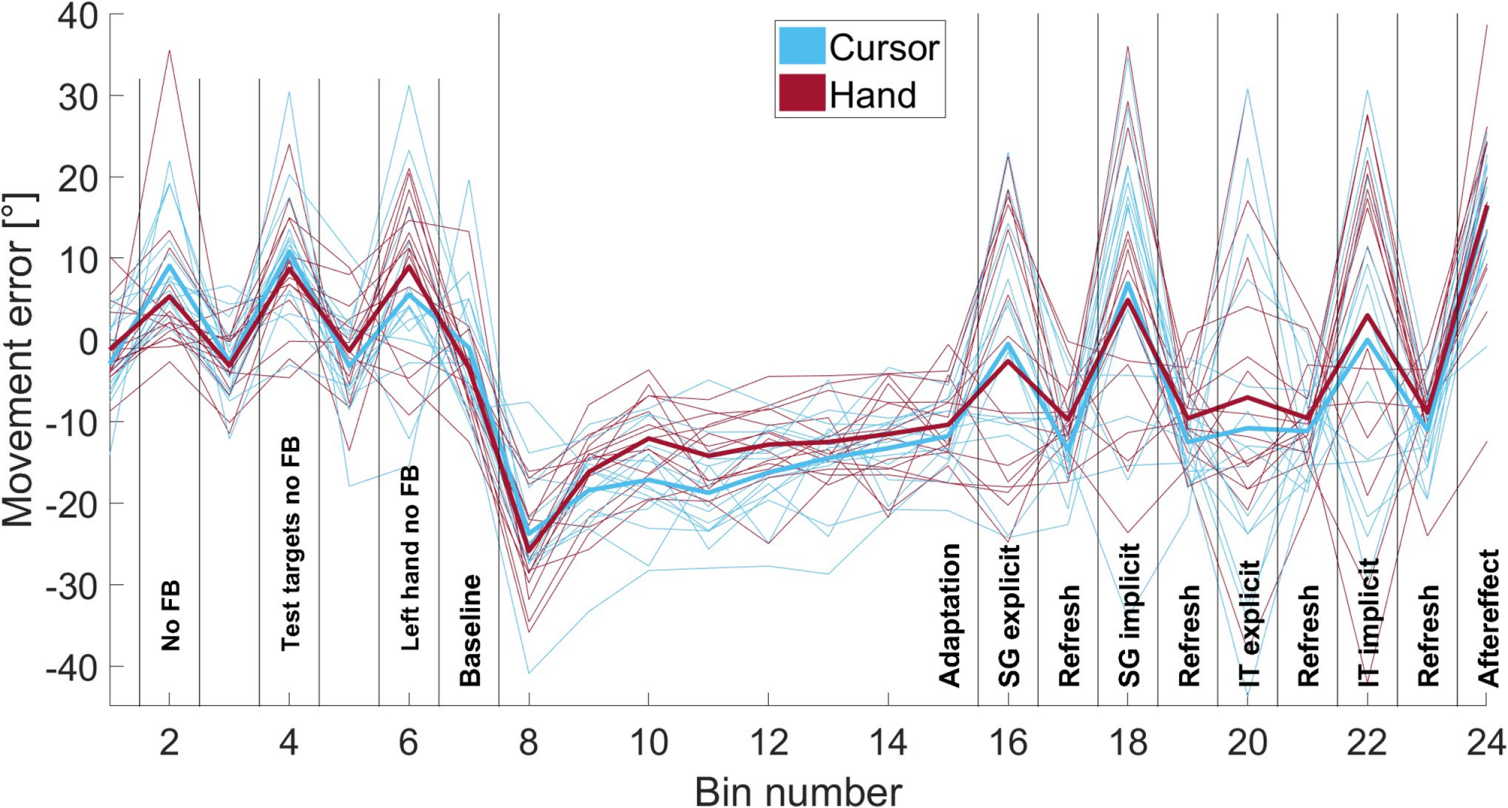
Angular movement errors of all bins: The figure displays the angular movement errors of all bins across 18 trials for groups C (blue) and H (red). The thick lines represent the mean values across subjects for each group, whereas the fine lines represent individual data points per participant. The bins include baseline bins (with and without feedback (FB), to the test targets and with the left hand), adaptation bins, spatial generalization bins (SG), intermanual transfer bins (IT), both explicit and implicit, refresh bins, and the measurement of the aftereffect.

Figure 1 shows the mean angular movement directions for both groups across all bins of each experimental block. Thin lines represent individual participants, whereas thick lines indicate group means (C in blue, H in red). The explicit bin is displayed before the implicit bin, despite randomization between participants. Baseline blocks without visual feedback exhibit high variability, likely owing to challenges in initiating movement via the yellow sphere. The participants received only distance-based feedback, leading to inconsistent starting positions across trials. This variability is attributed to participants starting at distances up to twice the starting sphere diameter, combined with the invisibility of the hand/cursor. A comparison of baseline values between groups revealed a significant effect of Bin (F(5, 128) = 18.082, *p* < 0.001, η² = 0.392) but no significant effect of Group (F(1, 28) = 0.057, *p* = 0.813) or the Bin × Group interaction (F(5, 128) = 1.006, *p* = 0.413).

### Adaptation

**Fig. 2:**
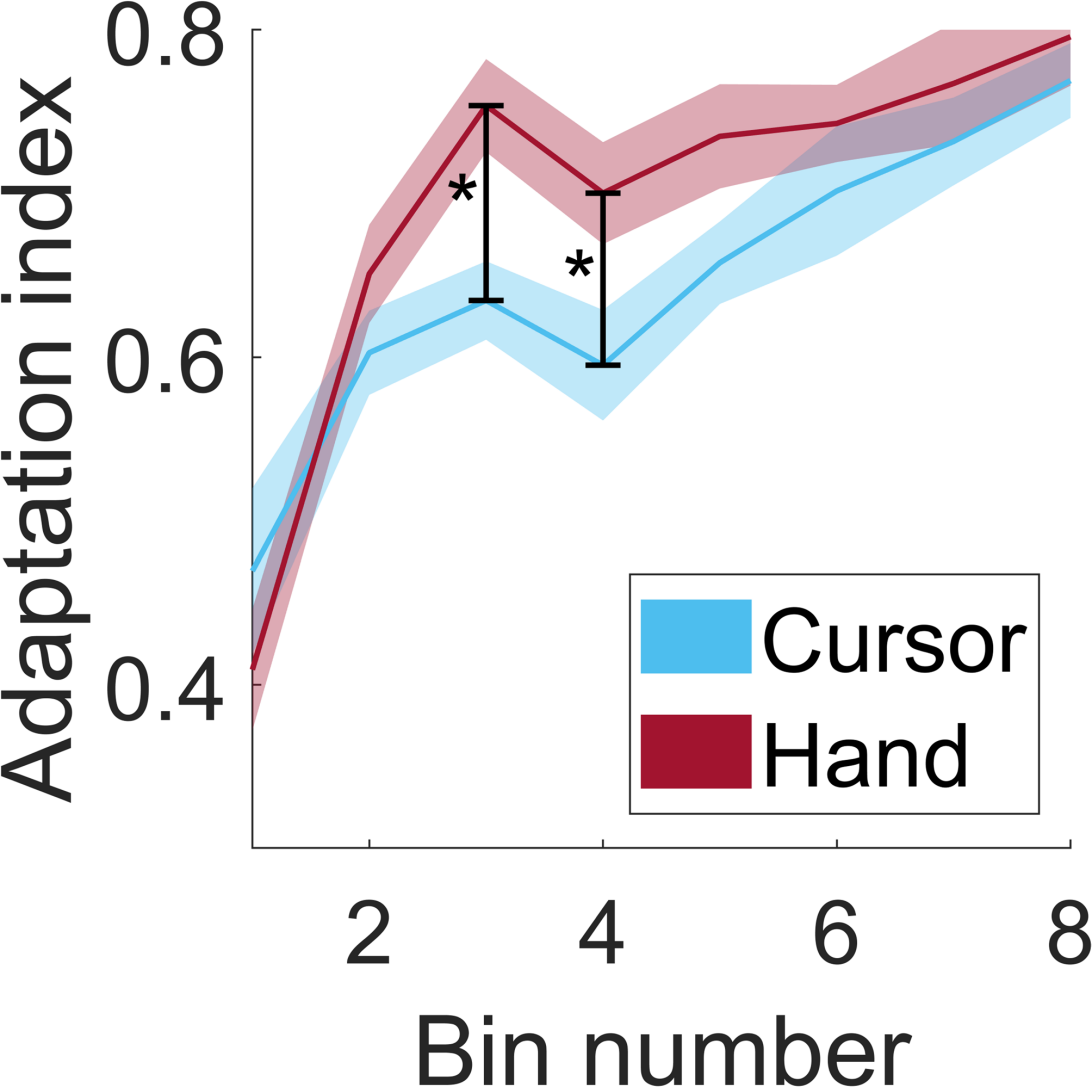
Mean adaptation indices of all bins and groups: The figure depicts the mean adaptation indices of all bins across 18 trials for groups C (blue) and H (red). The lines represent the mean values across subjects for each group, whereas the shaded areas represent the standard errors. Asterisks indicate significant differences between groups (p<0.05).

The mean adaptation indices for each bin of 18 trials are presented in Fig. 2. Both groups exhibit typical learning curves, characterized by an initial sharp increase in adaptation followed by a more gradual rise. Group H demonstrated a more pronounced increase at the onset of the adaptation block, although both groups ultimately reached comparable adaptation levels. This pattern is further supported by the statistical analysis. Two-way repeated-measures ANOVA, with the factors Group (C, H) and Bin (within-subject factor), revealed a significant main effect of Bin (F(6, 162) = 33.171, *p* < 0.001, η² = 0.542) and a significant Group × Bin interaction (F(6, 162) = 2.275, *p* = 0.041, η² = 0.075). No significant main effect was observed for the between-subjects factor Group (F(1, 28) = 2.839, *p* = 0.103). Post hoc comparisons revealed significantly lower adaptation in Group C than in Group H in the third bin (*p* = 0.003) and fourth bin (*p* = 0.030), with a marginal trend observed for the fifth bin (*p* = 0.068).

### Spatial generalization, intermanual transfer and aftereffect

**Fig. 3:**
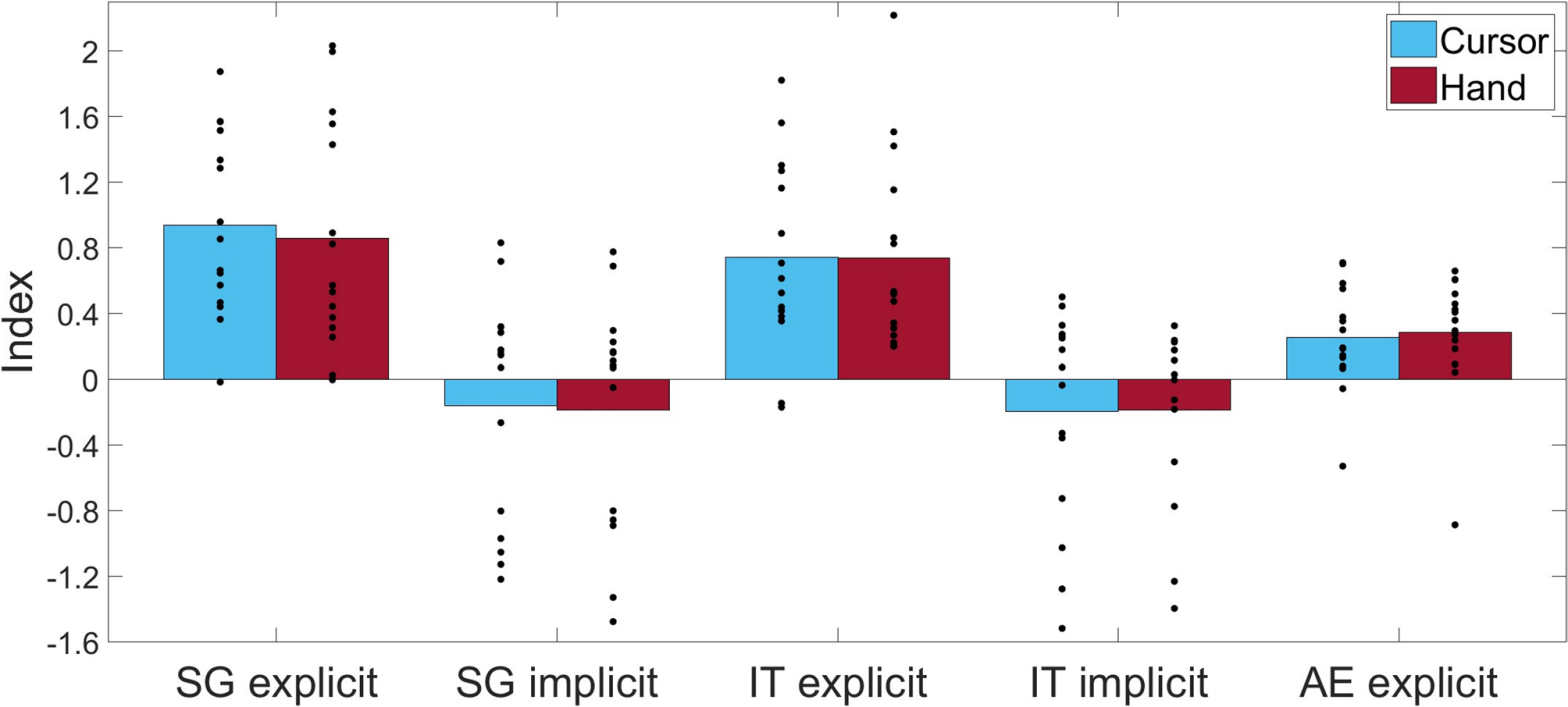
Generalization, intermanual transfer, and aftereffect: The figure presents the group mean values for the spatial generalization (SG), intermanual transfer (IM), and aftereffect (AE) indices for groups C (blue) and H (red). Individual data points are represented by dots for each group.

Figure 3 presents the results for spatial generalization, intermanual transfer, and aftereffects. The explicit tests of spatial generalization and intermanual transfer reflect total learning, encompassing both explicit and implicit components, as participants were informed of the cursor rotation and instructed to adjust their movements accordingly. In contrast, the explicit test of aftereffects reflects implicit learning, as participants were informed that the cursor rotation was not present. Both groups exhibit similar values across all posttests, and group comparisons revealed no significant differences [SG exp: t(28) = 0.357, *p* = 0.362; SG imp: t(28) = 0.107, *p* = 0.458; IT exp: U = 100, *p* = 0.624; IT imp: U = 98, *p* = 0.567; AE: U = 129, *p* = 0.512]. Further statistical analysis indicated that the values of the explicit tests and aftereffect measure were significantly different from zero for both the H and C groups. Specifically, for H: SG exp: t(14) = 4.785, *p* < 0.001, d = 1.236; IT exp: t(14) = 4.783, *p* < 0.001, d = 1.235; AE: t(14) = 2.969, *p* = 0.005, d = 0.767; and for C: SG exp: t(14) = 6.579, *p* < 0.001, d = 1.699; IT exp: t(14) = 4.921, *p* < 0.001, d = 1.270; AE: t(14) = 3.063, *p* = 0.004, d = 0.791. Owing to substantial variability observed in the baseline condition without visual feedback and posttest results, no further analyses were conducted on the posttest data.

### Sense of agency

**Fig. 4:**
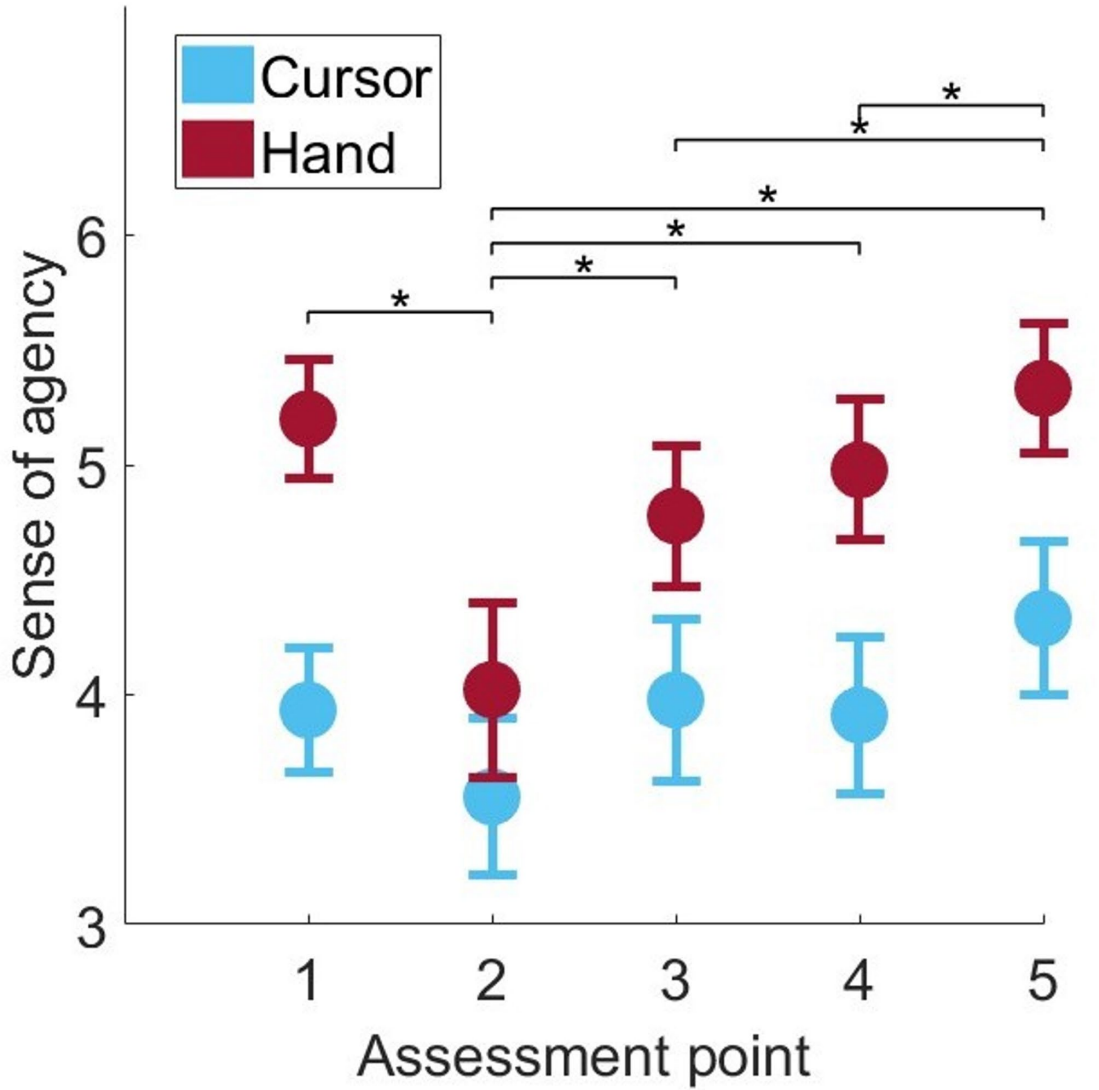
Sense of agency: The figure displays the sense of agency for groups C (blue) and H (red) per survey. The dots represent the across-subject means, and the error bars represent the standard errors. Asterisks indicate significant differences between assessment points (p<0.05).

The three statements from the agency questionnaire assess participants’ subjective perceptions of agency, ownership, and proprioception during task performance. The score ranges from 1 (lowest sense of agency) to 7 (highest), reflecting the extent to which participants experienced a sense of control over their movements. Figure 4 shows the mean values for all three statements across the five survey assessment points. This data is provided in supplementary information S1_Dataset and on OSF (https://osf.io/yb85q/). The first administration of the questionnaire took place after the baseline condition, in which no visual feedback rotation was applied. Subsequent administrations occurred after every two adaptation bins, corresponding to 36 movements. A notable decrease in the sense of agency values, particularly for the H group, was observed after the first two adaptation bins (second time point). Following this drop, the sense of agency values for both groups increased, with C consistently reporting a lower sense of agency than H.

ANOVA on the sense of agency scores, with the factors Group (C, H) and the within-subject factor Assessment point, revealed significant main effects for Assessment point (F(3, 97) = 10.441, *p* < 0.001, η² = 0.272) and Group (F(1, 28) = 5.332, *p* = 0.029, η² = 0.160). However, no significant interaction was found between Assessment pointand Group (F(3, 97) = 1.624, *p* = 0.182). Post hoc analyses of the main effect of Assessment pointrevealed significant differences between time point 2 and all other time points (*p* < 0.001). At assessment point 2, participants reported the lowest sense of agency, feeling least connected to their hand or the cursor. Assessment point 5 differed significantly from assessment points 2 (*p* < 0.001), 3 (*p* < 0.001), and 4 (*p* = 0.018) but not from assessment point 1 (*p* = 0.119). The sense of agency at assessment point 5 thus returned to a level comparable to that observed before the onset of the learning condition.

**Fig. 5:**
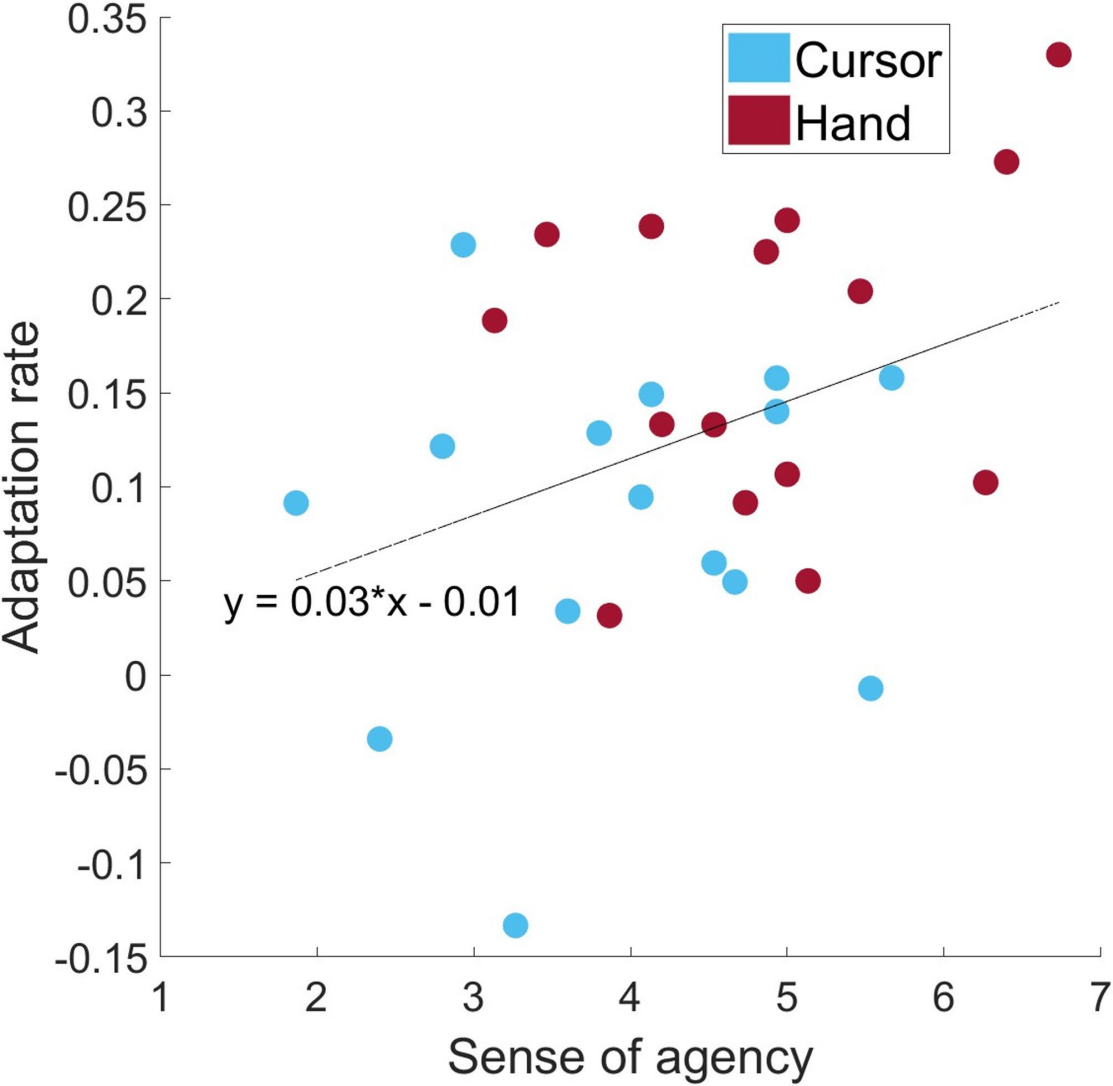
Correlation: The figure shows the correlation between the adaptation rate values and the mean sense of agency across all survey time points for the two groups, C (blue) and H (red).

To investigate the relationship between the speed of adaptation and the questionnaire data, we calculated the adaptation rate by determining the slope of a linear regression fitted to the first three bins of the adaptation index. A correlation analysis between the adaptation rate and the mean sense of agency across all survey time points revealed a moderate positive correlation (*r* = 0.360, *p* = 0.025), as shown in Fig. 5. Furthermore, a comparison of the adaptation rates between the two groups revealed that H presented a significantly higher adaptation rate than C did (t(28) = −2.757, *p* = 0.005, d = −1.007).

## Discussion

Our study aimed to investigate the role of visual feedback in visuomotor adaptation in VR, comparing a virtual hand (H group) to a cursor (C group). The primary finding was that the H group demonstrated faster adaptation than the C group, as indicated by a significant difference in the adaptation index. This finding supports the hypothesis that embodied feedback, which provides a stronger sense of agency, facilitates faster motor learning. However, no significant group differences were found in spatial generalization, intermanual transfer, or aftereffects. These results suggest that while visual feedback type influences adaptation speed, it does not necessarily impact other aspects of motor learning.

To address the first research question, the faster adaptation and greater sense of agency scores in the H group suggest that hand-based visual feedback enhances motor learning by strengthening the sense of agency. This aligns with findings that the controllability ratings of an avatar are significantly higher when it moves synchronously, actively, consistently, or resembles the physical body than when these factors are absent^39–42^. The participants in the H group presented a steeper initial adaptation curve and reported a stronger sense of agency than those in the C group did. The significant correlation between sense of agency and adaptation rate further supports the idea that a heightened sense of agency facilitates sensorimotor adaptation. Viewing a virtual hand rather than a cursor may provide a more intuitive and embodied representation of self-generated action, reinforcing the link between motor commands and visual outcomes. This enhanced perceptual-motor coupling could improve error correction, leading to faster adaptation. The initial drop in the sense of agency, particularly in the H group, might reflect a transient discrepancy between expected and perceived movement outcomes at the onset of adaptation. However, as learning progressed, the sense of agency recovered, mirroring adaptation stabilization. These results align with previous research suggesting that agency perception modulates motor learning efficiency^18^ and support the hypothesis that embodied visual feedback enhances adaptation.

Neuroimaging studies have demonstrated that motor learning relies on the integration of sensory inputs, including visual and proprioceptive signals^43,44^, within the parietal cortex and premotor areas^44–46^. Specifically, visual feedback can modulate activity in the primary motor cortex (M1), which is responsible for motor execution, and the posterior parietal cortex (PPC), which is crucial for spatial coordination and error correction. Additionally, the right angular gyrus and inferior parietal lobule – key regions for inferential processing and a sense of agency – have been implicated in the integration of sensory feedback^47^. Although effective multisensory integration is commonly associated with spatial alignment of stimuli^48,49^, previous research has demonstrated that integration can still occur in the presence of spatial discrepancies—particularly when stimuli are temporally congruent^43,50^. For example, Ernst and Banks^43^ showed that in visual–haptic integration, visual input can dominate the perceptual outcome. In our study, visual and motor signals were not spatially aligned due to the imposed visuomotor rotation. However, integration may still have been supported by the temporal coupling of these signals and the consistent causal relationship between motor actions and visual feedback. Furthermore, participants may have relied more heavily on visual information, suggesting a shift toward visual dominance during the adaptation process. The H group, benefiting from embodied feedback, may have exhibited enhanced connectivity between these regions, facilitating more efficient adaptation^9^.

The second research question examined whether adaptation with hand-based visual feedback would lead to greater spatial generalization, supporting context-independent learning^19–21,51^. However, our findings did not support this hypothesis, as no significant differences in spatial generalization were observed. This suggests that while hand-based feedback facilitated faster adaptation, it did not enhance generalization beyond the trained context. Previous research has proposed that the broader generalization observed in prism adaptation, compared with visuomotor adaptation, may result from differences in visual feedback (real hand vs. symbolic representation) and from differences in the error processing reference frame (body-centered vs. off-body-centered)^51^. Given that both groups exhibited comparable generalization patterns, our results suggest that the reference frame, rather than the visual representation type, is the critical factor influencing context-independent learning. Consistent with this idea, we propose that body-centered feedback in prism adaptation promotes generalized motor recalibration, whereas visuomotor adaptation remains rather context-dependent, regardless of feedback type. This interpretation aligns with previous findings showing that (implicit) generalization patterns are driven by body-related states rather than abstract contextual cues^52,53^.

To our knowledge, only a few studies have distinguished between explicit and implicit components of spatial generalization: these works^52–54^ have reported evidence for both implicit and explicit generalization. In contrast, our findings show strong explicit generalization (approximately 80% in both groups) but no implicit spatial generalization. However, a closer examination reveals an important consideration: previous studies have reported that spatial generalization in sensorimotor adaptation follows a Gaussian-shaped function, peaking at the trained target direction [e.g. 22,53]. This Gaussian profile has been linked to underlying neural tuning functions^6,55,56^.

Given this, we must consider that our aftereffect measure captures implicit “generalization” specifically at the trained target. When considering both our aftereffect and spatial generalization results, we observe implicit aftereffects at the trained location but no evidence of implicit generalization at the test targets positioned at ± 15°, ± 30°, and ± 45°. However, owing to the high variability in our no-feedback condition and substantial missing data in individual trials, a meaningful analysis of implicit generalization across different distances was not possible. Interpreting our findings within these limitations, our results align with those of previous studies showing that implicit adaptation is locally restricted, with approximately 30% of the learned rotation retained at the trained location. There are two possible explanations for our inability to detect implicit generalization beyond this point. First, implicit generalization may exhibit a Gaussian-shaped distribution, but our 3D paradigm was not sufficiently refined to capture it. Alternatively, we may be the first to measure implicit and explicit generalization in a 3D environment, particularly across different planes. This raises the possibility that previously observed generalization patterns are limited to the trained plane. Supporting this idea, recent findings demonstrate differences in adaptation rates between the horizontal, sagittal, and coronal planes^14^.

The third question concerns whether hand-based visual feedback strengthens implicit learning mechanisms, leading to larger aftereffects but reduced intermanual transfer. We found no support for this hypothesis in our data, as both groups presented similar aftereffects and intermanual transfer, suggesting that visual feedback type did not significantly influence the balance between explicit and implicit learning. This contradicts the findings of Wilke et al.^24^, who reported that a stronger internal attribution of sensory prediction errors led to greater aftereffects. One possible explanation for this discrepancy is methodological differences: Wilke et al.^24^ assessed recalibration via interspersed perceptual probe trials, whereas we measured aftereffects at the end of the adaptation phase. A more plausible interpretation, however, is that Wilke et al.’s data suggest that even substantial increases in perceived errors of 10° result in only small changes in recalibration of 2°, implying that the difference between hand- and cursor-based feedback may not have been large enough to yield measurable effects in our highly variable aftereffect data. Furthermore, our results contrast with those of Ramos et al.^17^, who reported greater aftereffects in virtual reality (VR) than in physical prism adaptation. However, their study compared VR conditions to non-VR conditions, introducing potential confounding factors.

Moreover, our data provide the first evidence of the intermanual transfer of visuomotor adaptation in 3D movements within a VR environment. We observed explicit but not implicit transfer, which is consistent with previous findings in 2D settings, where intermanual transfer has been attributed primarily to explicit adaptation^27,37,38^. These findings suggest that the mechanisms underlying visuomotor adaptation in 2D movements may also apply to our 3D setup. Notably, we found a large overall intermanual transfer (explicit plus implicit) of nearly 80%, whereas previous 2D studies reported transfer rates of approximately 30–50%^57^ or 50–65% in our own prior work^27^. However, because the transfer magnitude is influenced by numerous factors, such as movement direction (dominant to nondominant hand or vice versa^57–59^;), handedness^60–62^ and measurement methods, we remain cautious in drawing direct comparisons between intermanual transfer in 2D and 3D settings.

Previous research comparing visuomotor adaptation in immersive VR with conventional 2D training has reported either a reduced^17^ or an increased reliance on explicit processes^16^. Juliano et al.^63^ further examined differences between VR and 2D visuomotor adaptation, finding reduced explicit adaptation in VR, which they attributed to the higher cognitive load imposed by the immersive environment. However, since both groups in our study trained within a VR setting, direct comparisons with these previous findings are challenging. Nevertheless, it is possible that the hand-feedback group experienced a mitigated cognitive load effect, as suggested by their faster adaptation, which may indicate a greater reliance on explicit strategies^28^. However, our findings on the effects of visual feedback type – hand-based versus cursor-based – on spatial generalization, intermanual transfer, and aftereffects suggest that the specific form of feedback does not play a decisive role in modulating the balance between explicit and implicit learning.

In conclusion, our study demonstrates that hand-based visual feedback accelerates visuomotor adaptation of 3D movements in VR, likely due to an increased sense of agency. However, this enhanced adaptation did not translate into broader generalization, altered intermanual transfer, or a larger aftereffect. These findings suggest that while the embodiment of visual feedback modulates adaptation speed, it does not fundamentally alter the mechanisms governing implicit and explicit learning. These insights carry important implications for the design of VR-based interventions in both clinical rehabilitation and athletic training. VR enables intensive, adaptive practice environments that can enhance neuroplasticity and support recovery in patients with motor impairments^64,65^. Our results, highlighting the influence of feedback modality on motor adaptation and intermanual transfer, are especially relevant for designing targeted training protocols in stroke rehabilitation^25^ or upper-limb prosthesis use^66^. They also support the integration of embodied feedback to optimize sensorimotor learning. Beyond clinical contexts, these findings may improve VR-based training in sports, where individualized, high-repetition, low-risk environments can accelerate skill acquisition^67,68^. By identifying feedback types that support efficient motor learning, our study contributes to evidence-based development of VR training tools across health and performance domains.

## Methods

### Participants

Thirty right-handed volunteers (18–30 years old) participated in the study. Although the total sample size of 30 participants (15 per group) is relatively modest, this limitation is mitigated by the high number of trials per participant. This design increases the reliability and statistical power of within-subject analyses. It also aligns with established practices in perceptual and sensorimotor research, where dense within-subject data can compensate for smaller between-subject samples^43^. The participants’ handedness was evaluated by calculating the laterality quotient by Oldfield^69^. None had a history of neurological disorders or prior experience with visuomotor adaptation research. The participants were randomly assigned to two test groups, which were age- and sex-matched. Handedness was confirmed via the Edinburgh Handedness Inventory^69^. The study was approved by the local Ethics Committee and conducted in accordance with the Declaration of Helsinki. All participants provided written informed consent.

### Set-up

**Fig. 6:**
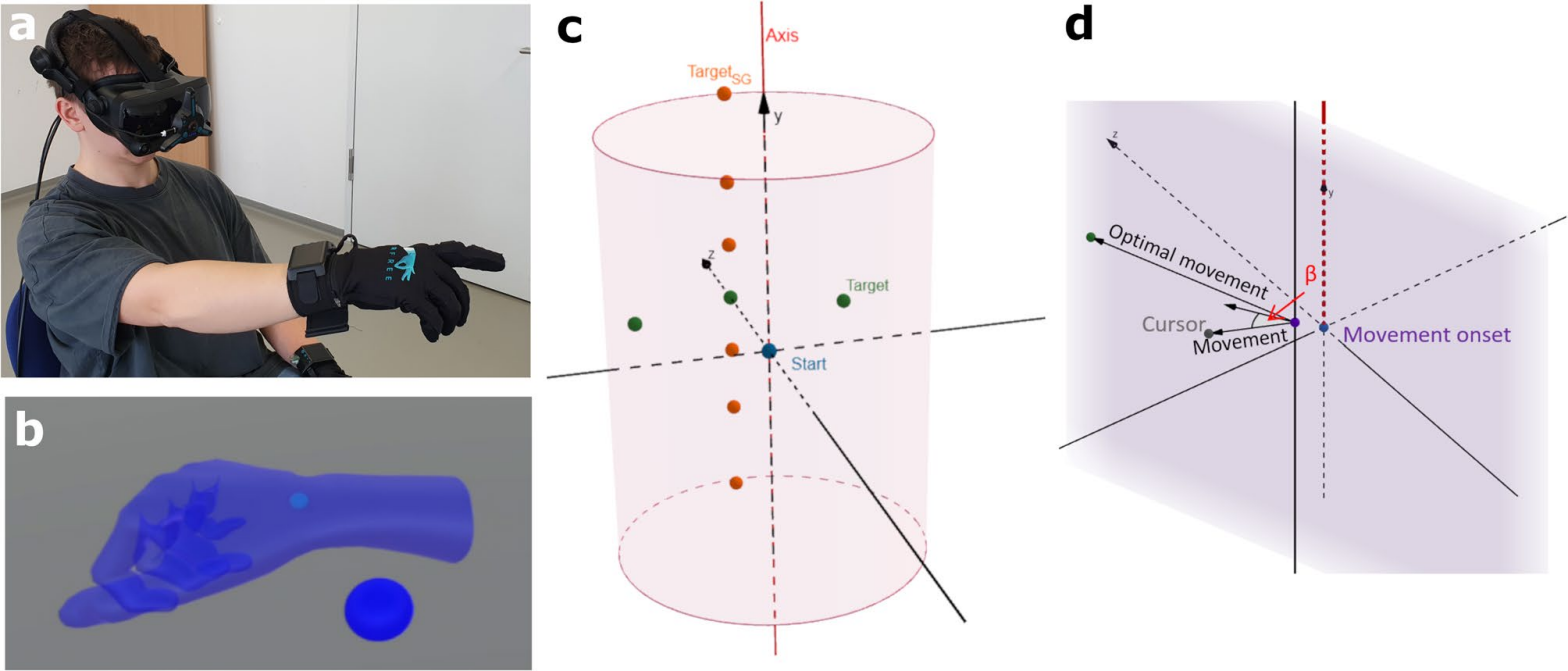
Experimental setup (a): The figure displays the subject wearing a VR headset and gloves seated in a chair. Subject’s view in VR (b): The subject’s view in VR includes the starting sphere displayed in dark blue. The subject was able to see either the blue hand with the turquoise cursor at the center of the wrist or solely the cursor. Experimental task (c): The experiment involved a rotation of 40 degrees. The starting sphere is depicted in blue, the training targets are depicted in green, and the test targets for evaluating spatial generalization are depicted in orange. Data analysis (d): Data were analyzed by measuring the movement error β, which was calculated as the deviation between the actual movement and its perpendicular projection onto a plane. This plane extends between the movement onset and the target along the rotational axis direction.

As illustrated in Fig. 6a, the participants were seated in an armless chair to minimize extraneous body movements and maximize the range of motion for the upper limbs. A virtual reality (VR) environment was implemented via Valve Index^®^ VR goggles, SteamVR 2.0 base stations, and Sensoryx VRfree^®^, a mobile tracking system comprising a head module and two wireless gloves. The VRfree^®^ system recorded and transmitted hand position data at a frequency of 100 Hz, with a spatial accuracy of 0.3 mm and a precision of 0.1 mm.

To ensure consistent alignment of the virtual coordinate system across participants, the position of the chair and the initial orientation of the VR headset were standardized. The coordinate system was defined by the hardware, with the x-axis representing the sagittal axis, the y-axis representing the vertical transverse axis, and the z-axis representing the frontal axis. The use of wireless gloves allowed participants unrestricted hand movement.

The VR environment presented a virtual laboratory. The hand position, tracked via the VR gloves, was visually represented in the virtual space either as a hand (without an arm) or as a cursor at the wrist, depending on the experimental conditions (see Fig. 6b). The cursor, with a diameter of 0.5 cm, was positioned precisely at the output position of the VR glove. The participants’ real arms were fully occluded by the VR headset to ensure exclusive reliance on virtual visual feedback.

VR software was developed via the Unity™ Game Engine and controlled via a Windows-based application implemented with C# WPF MVVM Prism. The system operated on a standalone PC configuration, as no client-server architecture was needed. The experimental data, including the test and result files, were recorded in JSON format.

### Task

Each trial involved the sequential presentation of a starting sphere followed by one of three possible target spheres, as illustrated in Fig. 6c. The starting sphere had a diameter of 3 cm, whereas the target spheres had a diameter of 2 cm. The target spheres were positioned 15 cm away from the starting sphere within the xz-plane and spaced 45° apart. To assess spatial generalization after adaptation, six additional test targets were introduced. These targets were vertically displaced along the y-axis while maintaining the same x- and z-coordinates as the central training target. They were positioned at angles of ± 15°, ± 30°, and ± 45° relative to the xz-plane (see Fig. 6c for an overview).

A trial was initiated when the participant moved the cursor to the center of the starting sphere. Once the cursor remained within a predefined proximity threshold (< 4 times the radius of the starting sphere) for 500 ms, the starting sphere changed color from blue to green. After an additional 1000 ms, the starting sphere disappears, and a target sphere appears simultaneously. The target sphere remained visible for 1250 ms, defining the movement duration of each trial. The target sphere subsequently disappeared, and the starting sphere reappeared, signaling the beginning of the next trial. The participants were instructed to execute rapid, straight movements toward the target without making corrective adjustments to their aiming direction.

To prevent fatigue-related performance decreases, participants were given breaks every 6 to 18 trials. The maximum of 18 consecutive trials was established on the basis of pilot testing to minimize exhaustion effects. The break durations were fixed but varied between 15 s and 60 s, depending on the need for additional task instructions. The participants were instructed to rest their hand on their thigh during breaks without removing the VR headset.

Certain trials were conducted without visual feedback. In these trials, all visual feedback was removed upon target sphere presentation, requiring participants to complete the movement without sight of their hand or cursor. During the return movement to the starting position, a semitransparent yellow sphere was displayed to indicate the distance between the participant’s hand (or cursor) and the starting sphere, facilitating repositioning.

### Experimental design

The participants were randomly assigned to one of two groups: the H group (aged 24.0 ± 2.1 years; 9 females and 6 males), in which the participants received visual feedback in the form of a virtual hand, and the C group (aged 24.4 ± 2.5 years; 9 females and 6 males), in which only a cursor was visible. The experimental protocol is outlined in Table 1. The participants first completed 45 familiarization trials with veridical feedback, followed by 78 baseline trials for both the right and left hands, as well as for training and test targets. During the baseline trials, visual feedback was either undistorted or absent. This was followed by an adaptation block of 144 trials, during which the visual feedback was rotated 40° counterclockwise (CCW) around the y-axis (Fig. 1C). The transformation was applied according to the following rotation matrix:

**Table 1:** Experimental protocol: visual feedback (FB) was either absent (-), veridical (0°), or rotated (40°). Unless otherwise specified, trials were conducted with the right hand. The sequence of explicit and implicit testing was alternated among participants.

Blockname	# of trials	Visual FB
Familiarization right and left hand	45	0 °
Baseline	6	0 °
Baseline no FB	18	-
Baseline	6	0 °
Baseline test targets	18	0 °
Baseline	6	0 °
Baseline left hand no FB	18	-
Baseline	6	0 °
Adaptation	144	40 °
Test targets explicit/implicit	18	-
Refresh	18	40 °
Test targets implicit/explicit	18	-
Refresh	18	40 °
Transfer left hand explicit/implicit	18	-
Refresh	18	40 °
Transfer left hand implicit/explicit	18	-
Refresh	18	40 °
Washout	18	-

1 $$\:\left(\begin{array}{c}x{\prime\:}\\\:y{\prime\:}\\\:z{\prime\:}\end{array}\right)=\left(\begin{array}{ccc}\begin{array}{c}{{n}_{1}}^{2}\left(1-\text{cos}\delta\:\right)+\text{cos}\delta\:\\\:{n}_{2}{n}_{1}\left(1-\text{cos}\delta\:\right)+{n}_{3}\text{sin}\delta\:\\\:{n}_{3}{n}_{1}\left(1-\text{cos}\delta\:\right)-{n}_{2}\text{sin}\delta\:\end{array}&\:\begin{array}{c}{n}_{1}{n}_{2}\left(1-\text{cos}\delta\:\right)-{n}_{3}\text{sin}\delta\:\\\:{{n}_{2}}^{2}\left(1-\text{cos}\delta\:\right)+\text{cos}\delta\:\\\:{n}_{3}{n}_{2}\left(1-\text{cos}\delta\:\right)+{n}_{1}\text{sin}\delta\:\end{array}&\:\begin{array}{c}{n}_{1}{n}_{3}\left(1-\text{cos}\delta\:\right)+{n}_{2}\text{sin}\delta\:\\\:{n}_{2}{n}_{3}\left(1-\text{cos}\delta\:\right)-{n}_{1}\text{sin}\delta\:\\\:{{n}_{3}}^{2}\left(1-\text{cos}\delta\:\right)+\text{cos}\delta\:\end{array}\end{array}\right)\left(\begin{array}{c}x\\\:y\\\:z\end{array}\right)+\left(\begin{array}{c}{s}_{1}\\\:{s}_{2}\\\:{s}_{3}\end{array}\right)$$

The transformed, distorted position (x′, y′, z′) was computed via the rotation matrix that incorporated the normal vector of the rotation axis (n1,n2,n3), the rotation angle δ, the undistorted position (x, y, z), and the position of the starting sphere (s1, s2, s3).

For the H group, in addition to the positional perturbation, the orientation of the virtual hand was also rotated 40° counterclockwise (CCW) around the output position, following the transformation:

2 $$\:\left(\begin{array}{c}{o}_{x}\\\:{o}_{y}\\\:{o}_{z}\end{array}\right)=\left(\begin{array}{c}\alpha\:+{n}_{1}\delta\:\\\:\beta\:+{n}_{2}\delta\:\\\:\gamma\:+{n}_{3}\delta\:\end{array}\right)$$

The recalculated orientation relative to the x-, y-, and z-axes is represented by the vector (ox, oy, oz). The angles α, β, and γ correspond to the roll around the x-axis, the pitch around the y-axis, and the yaw around the z-axis, respectively, in the undistorted orientation.

The participants were not informed about the visuomotor perturbation; they were only informed that the task would become more challenging. Following the adaptation phase, several testing blocks were conducted without visual feedback to assess spatial generalization and intermanual transfer. The spatial generalization test involved movements to the test targets, whereas intermanual transfer was assessed via the left hand. To distinguish between explicit and implicit generalization and transfer, participants were instructed either to “use what was learned during adaptation” or to “refrain from using what was learned and perform movements as during baseline.” Each testing block consisted of 18 trials, and the order of the explicit and implicit tests was randomized. To minimize the decay of adaptation effects, a refresh block of 18 trials, identical in setting to the adaptation phase, was introduced after each testing block. Finally, aftereffects were assessed in a block of 18 trials without visual feedback, during which participants were instructed to perform movements as they did during baseline.

In addition to the behavioral data, the sense of agency was assessed at five time points via a questionnaire. After the baseline phase and at every second break during adaptation, the assessment of the participants was conducted via a 7-point Likert scale, where 1 indicated full disagreement and 7 represented full agreement with the statements. The participants rated three items from Ziadeh et al.^70^ concerning their sense of agency, ownership, and proprioception on this scale.

### Data processing

The position data were processed and analyzed via MATLAB. Since the data recording frequency was not constant, the data were interpolated via a piecewise cubic Hermite interpolating polynomial (PCHIP). To obtain a smoothed velocity profile, a Savitzky‒Golay filter was applied. On the basis of this velocity profile, movement onset was defined as the time point at which the velocity exceeded 10 cm/s.

The movement direction vector was determined using the positions at onset and 150 ms after onset, which were obtained through data interpolation. Trials where the onset velocity criterion was not met were excluded (38 trials). A reaction time threshold of 1 s was imposed, but no trials exceeded this limit. To ensure that movements originated from the intended starting position, trials were discarded if the onset position deviated more than 10 cm from the starting sphere (91 trials). Trials were also excluded if the displacement between onset and 150 ms later was less than 2 cm, indicating insufficient movement execution (176 trials). Additionally, trials were visually inspected for excessive deviation between the movement direction and the onset-to-target vector (threshold: 40° for unperturbed blocks, perturbation angle + 10° for perturbed blocks). This identified and excluded trials where data transfer issues between the glove and the computer caused abrupt discontinuities or jumps in the recorded data (363 trials). In total, 668 out of 11,520 trials were excluded from the final analysis.

Movement accuracy was quantified via an error angle, defined as the intersection angle between a straight-line trajectory and a reference plane. The trajectory was represented by a vector from the position at movement onset to the position 150 ms later. The reference plane was defined by two vectors: one extending from the movement onset position to the target and another aligned with the rotation axis (y-axis), as shown in Fig. 6d. This approach allowed for the isolation of movement errors in the perturbation direction while excluding errors in the vertical dimension. The entire list of all mean movement errors of each bin of 18 trials for each participant and all groups (C, H) is provided as supplementary information S2_Dataset and on OSF (https://osf.io/yb85q/).

Indices were calculated for adaptation, explicit and implicit generalization, transfer, and aftereffect trials to ensure comparability with other studies and a parallel experiment employing a 60° perturbation. This approach enhances the broader applicability of the results.

3 $$\:Adaption:\frac{(ADAP-BL)-ROT}{-ROT}$$

4 $$\:Explicit:\frac{({EXP}-{BL}_{x})-ROT}{\:({ADAP}_{L}-BL)-ROT}$$

5 $$\:Implicit:\frac{{IMPL}-{BL}_{x}}{\:({ADAP}_{L}-BL)-ROT}$$

6 $$\:Aftereffect:\frac{{AE}-{BL}_{noFB}}{\:({ADAP}_{L}-BL)-ROT}$$

The adaptation index for each bin was calculated via the mean adaptation error (ADAP), the mean baseline error of all trials with visual feedback (BL), and the rotation angle (ROT). The explicit index was normalized by dividing the explicit block (EXP) by the corresponding baseline (BL_x_) for test targets or the left hand without visual feedback, with the denominator being the mean of the last 18 adaptation or refresh trials (ADAP_L_). The implicit index was derived from implicit trials (IMPL), whereas the aftereffect index was based on aftereffect trials (AE), both of which use the same denominator as the explicit index. BL_noFB_ represents the baseline without feedback for training targets. For the adaptation and aftereffect indices, the set rotation angle (ROT) was used. Since the rotation angle varies with the target distance from the starting sphere along the rotation axis, this adjusted angle was used for spatial generalization calculations.

Baseline and adaptation data were analyzed via two ANOVAs with the between-subject factor Group (H, C) and the within-subject factor Bin. Explicit and implicit spatial generalization, intermanual transfer, and aftereffect indices were each analyzed via separate one-factor ANOVAs with Group as the between-subject factor. Huynh–Feldt adjustments were applied when necessary to correct for variance heterogeneity. Effect sizes for significant differences are reported as Eta-squared (η²). Significant effects were examined via Fisher’s LSD post hoc tests. Generalization and aftereffects were compared via independent-samples Student’s t tests or, when normality was violated, Mann‒Whitney U tests, with effect sizes reported as Cohen’s d. Correlations between the adaptation rate and sense of agency were assessed via Pearson’s correlation coefficient (R), and for significant correlations, adaptation rates between groups were compared via an independent-samples t test. All the statistical analyses were conducted in SPSS (Version 27.0, IBM Corp., Armonk, NY).

## Electronic supplementary material

Below is the link to the electronic supplementary material.

Supplementary Material 1

Supplementary Material 2

## Data Availability

All the datasets analyzed during the current study are available in the online repository (https://osf.io/yb85q/).
